# Antimicrobial Resistance of and Genomic Insights into Pasteurella multocida Strains Isolated from Australian Pigs

**DOI:** 10.1128/spectrum.03784-22

**Published:** 2023-01-18

**Authors:** Alec Truswell, Tanya J. Laird, Suzanna Jones, Mark O’Dea, John Blinco, Rebecca Abraham, Daniel Morison, David Jordan, David J. Hampson, Stanley Pang, Sam Abraham

**Affiliations:** a Antimicrobial Resistance and Infectious Diseases Laboratory, Harry Butler Institute, Murdoch University, Murdoch, Western Australia, Australia; b ACE Laboratory Services, Bendigo, Victoria, Australia; c New South Wales Department of Primary Industries, Wollongbar, New South Wales, Australia; Innovations Therapeutiques et Resistances

**Keywords:** *Pasteurella multocida*, pigs, Australia, antimicrobial resistance, genomic comparisons

## Abstract

Infection with Pasteurella multocida represents a significant economic threat to Australian pig producers, yet our knowledge of its antimicrobial susceptibilities is lagging, and genomic characterization of P. multocida strains associated with porcine lower respiratory disease is internationally scarce. This study utilized high-throughput robotics to phenotypically and genetically characterize an industry-wide collection of 252 clinical P. multocida isolates that were recovered between 2014 and 2019. Overall, antimicrobial resistance was found to be low, with clinical resistance below 1% for all tested antimicrobials except those from the tetracycline class. Five dominant sequence types, representing 64.8% of all isolates, were identified; they were disseminated across farms and had previously been detected in various animal hosts and countries. P. multocida in Australian farms remain controllable via current antimicrobial therapeutic protocols. The identification of highly dominant, interspecies-infecting strains provides insight into the epidemiology of the opportunistic pathogen, and it highlights a biosecurity threat to the Australian livestock industry.

**IMPORTANCE** Pasteurellosis is rated by the World Animal Health Organisation (OIE) as a high-impact disease in livestock. Although it is well understood in many host-disease contexts, our understanding of the organism in porcine respiratory disease is limited. Given its high frequency of involvement in porcine respiratory disease complex (PRDC), it is important that we are aware of its antimicrobial susceptibilities so that we can respond quickly and appropriately with antimicrobial therapy. Genetic insights about the organism can help us to better understand its epidemiology and inform our biosecurity practices and prophylactic management.

## INTRODUCTION

Infectious respiratory disease is the primary health-related challenge facing the swine industry worldwide and is estimated to cost producers approximately Aust$2.80 per untreated pig (1). The disease typically results from several pathogens acting in synergy, in a syndrome referred to as porcine respiratory disease complex (PRDC) (2). While PRDC can vary greatly in terms of the infectious agents involved, its pathogenesis remains rather formulaic; a primary pathogen initiates disease and impairs respiratory defenses, facilitating colonization of the lower respiratory tract by secondary (opportunistic) pathogens and subsequent exacerbation of the disease state (3). It is this complication of disease by secondary pathogens that is more frequently associated with significant economic loss, making these secondary pathogens key targets for controlling losses due to porcine respiratory diseases.

Studies analyzing the frequency of component pathogens contributing to PRDC infections have implicated Pasteurella multocida as the most commonly detected secondary pathogen (4, 5). P. multocida is a Gram-negative coccobacillus that constitutes part of the normal flora of the upper respiratory tract, with the capacity to opportunistically contribute to disease in several livestock species (6). Pneumonia is a common component of PRDC, and the participation of P. multocida in PRDC tends to skew disease from mild pneumonia toward either suppurative bronchopneumonia with occasional pleuritis or, in some cases, pleuropneumonia preceded by infection with swine influenza virus (2). The repercussions of pneumonic pasteurellosis are wide-ranging, with a gradient from reduced weight gain to death, and economic impacts are inevitable (7).

A variety of schemes have been employed for subspecific differentiation of P. multocida. The most enduring approach is serotyping based on capsular polysaccharide and lipopolysaccharide antigens, for which molecular methods also have been developed (8). More recently, multilocus sequence typing (MLST) based on allelic profiling has been employed (9). Capsular serogroup-based typing is the only form of classification thus far to have identified a clear predilection of certain strains to be causative or associated with specific diseases. Of particular relevance is the association between capsular serogroup (or genotype) A and PRDC (7). Although it is well understood in many host-disease contexts, there is limited high-resolution genomic characterization of P. multocida in cases in which it has been associated with porcine pneumonia (10). Of the 1,741 P. multocida isolates characterized by MLST that were in the PubMLST database at the time this study was completed, <150 pertain to cases of porcine lower respiratory tract disease, with no new submissions since 2016 (11). This paucity of genetic data limits opportunities to understand strain epidemiology, pathogenicity, and affiliation with antimicrobial resistance genes (ARGs), information of great value to disease management strategies.

This lack of characterization of PRDC-associated P. multocida strains also extends to their phenotypic properties. Surveillance of antimicrobial resistance (AMR) among PRDC-associated P. multocida strains is conducted infrequently, making it difficult to identify emerging or expanding resistances. The infrequent occurrence of AMR in P. multocida strains documented in a previous Australian surveillance study (12) is not a guarantee of ongoing stability, as exemplified by other bacterial species in production animals for which the occurrence of nonsusceptibility has stealthily increased or new resistances have arisen by various means, including mutation and horizontal gene transfer (13). Together, the experiences with PRDC point to a need for a form of surveillance that relies on more isolates being assessed more comprehensively and more often. A scheme to achieve this involves one or more diagnostic laboratories proactively accumulating isolates and then submitting them to a central facility for detailed analysis. More advanced capabilities of the receiving laboratory allow genomic and phenotypic scrutiny of panels of isolates to be performed rapidly, using standardized assays under standard conditions and on a large scale. Electronic reporting then makes timely interventions at the herd level a reality.

This study aimed to clarify the genetic and phenotypic context of Australian porcine P. multocida strains by using a 5-year (2014 to 2019) collection of isolates that had been identified as contributors to respiratory tract infections (Table 1) and a robotic antimicrobial susceptibility platform (RASP) adapted to handle large libraries of isolates. Given the “closed” nature of the Australian national herd, with there being no live pig imports for over 30 years (14), and conservative use of antimicrobials (15), we hypothesized that genetically distinct isolates would be found circulating within and between Australian farms, with these harboring resistance only to antimicrobials of low importance in human medicine (16).

**TABLE 1:** Number of Pasteurella multocida isolates used in the study and their respective sites of isolation (*n* = 252)

Site(s) of isolation	No. of isolates	No. of farms
Abdomen	3	3
Brainstem	1	1
Lung	226	62
Lung and heart	1	2
Gut	1	1
Heart	5	2
Hock	2	1
Lymph node and lung	1	1
Nasal cavity	1	1
Spleen	1	1
Tendon sheath	1	1
Trachea	4	1
Unknown	4	3

## RESULTS

### Phenotypic AMR.

Antimicrobial susceptibility data from broth microdilution assays generally identified <1% of isolates as being resistant to each antimicrobial, with the exception of chlortetracycline (22.9% resistant) and tetracycline (23.3% resistant) (Table 2). Low rates of resistance were seen against ampicillin (0.4%), florfenicol (0.4%), and trimethoprim-sulfamethoxazole (0.8%).

**TABLE 2:**
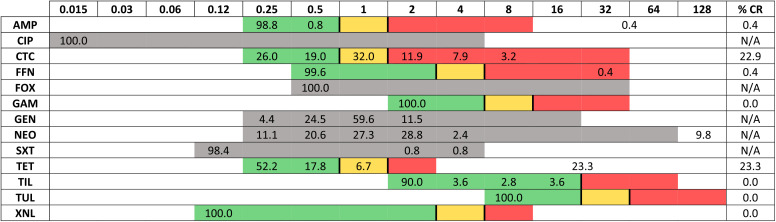
Distribution of MICs for 252 Pasteurella multocida isolates collected from Australian pigs between 2014 and 2019^*a*^

a The percentage of isolates is shown for each drug concentration. Isolates were classified as clinically resistant based on CLSI guidelines. Shaded areas indicate the range of dilutions evaluated for each antimicrobial; green, yellow, and red shading indicates susceptible, intermediate, and resistant concentrations, respectively, and gray shading is used when no CLSI breakpoints are available. Data outside the shaded areas (white area) represent cases in which the MIC exceeded the tested concentration range. AMP, ampicillin; CIP, ciprofloxacin; CTC, chlortetracycline; FFN, florfenicol; FOX, cefoxitin; GAM, gamithromycin; GEN, gentamicin; NEO, neomycin; SXT, trimethoprim-sulfamethoxazole; TET, tetracycline; TIL, tilmicosin; TUL, tulathromycin; XNL, ceftiofur.

### Genotypic AMR.

A total of 13 ARGs were detected across the collection of isolates, associated with the provision of phenotypic resistance to a range of antimicrobial classes, including aminoglycosides [*ant(9)-Ia*, *aph(3″)-Ib*, *aph(3)-Ia*, and *aph(6)-Id*], β-lactams (*bla*_ROB-1_), folate pathway antagonists (*dfrA14* and *sul2*), macrolides [*erm*(A)], phenicols (*floR*), and tetracyclines [*tet*(B) and *tet*(Y)]. Overall, a low rate of carriage of ARGs was seen, with no ARGs detected in 75.5% of screened isolates (see Table S2 in the supplemental material).

### Virulence factors.

Of the 23 virulence factors screened in this study, 98.9% of isolates were carrying ≥15 (Table 3). All isolates carried at least one virulence factor from the iron acquisition, adhesin, protectin, and superoxidase dismutase function groups. High levels of carriage in isolates were seen for virulence factors from the hyaluronidase and sialidase function groups, i.e., 75.1% for *pmHAS* and 65.2% for *nanH* (not mutually exclusive with *nanB* at 18.6%). No observable difference was seen regarding virulence factor carriage by respiratory tract-derived versus non-respiratory tract-derived isolates.

**TABLE 3:** Virulence factor carriage by 252 Pasteurella multocida isolates obtained from Australian pigs between 2014 and 2019

Function and gene	Detection rate (%)
Iron acquisition	
*exbB*	100.0
*exbD*	100.0
*Fur*	99.6
*hgbA*	96.4
*hgbB*	93.3
*tbpA*	0.0
*tonB*	99.6
Adhesins	
*fimA*	87.0
*hsf-1*	90.1
*hsf2*	34.4
*ptfA*	100.0
*tadD*	60.1
*pfhA*	62.3
Protectins	
*oma87*	100.0
*ompA*	100.0
*ompH*	22.9
*plpB*	36.0
Sialidase	
*nanB*	18.6
*nanH*	65.2
Superoxide dismutase	
*sodA*	100
*sodC*	99.6
Hyaluronidase	
*pmHAS*	75.1
Toxin	
*toxA*	0.0

### Strain typing.

The whole-genome sequencing (WGS) data were used to identify capsular and lipopolysaccharide (LPS) genotypes. Capsular genotype A represented 75% of the isolates, followed by 23% of the isolates with capsular genotype D, 1% of the isolates with capsular genotype F, and 1 untypeable isolate. For LPS genotyping, isolates belonged to either L1 (8%), L3 (62%), or L6 (30%) genotype. Among the 252 P. multocida isolates, 31 unique sequence types (STs) were represented in various proportions. The most dominant STs included ST 124 (27.8%), ST 9 (15.5%), ST 167 (7.9%), ST 50 (7.5%), ST 20 (6.7%), and ST 151 (6.7%), while the other 24 STs each represented less the 5% of the isolates (see Table S3). Of the 252 isolates that underwent sequence typing, 26 (10%) did not belong to previously registered Rural Industries Research and Development Corp. (RIRDC) STs and thus were registered in the PubMLST database as 14 new RIRDC STs (STs 379 to 392).

### Phylogenetics.

**FIG 1:**
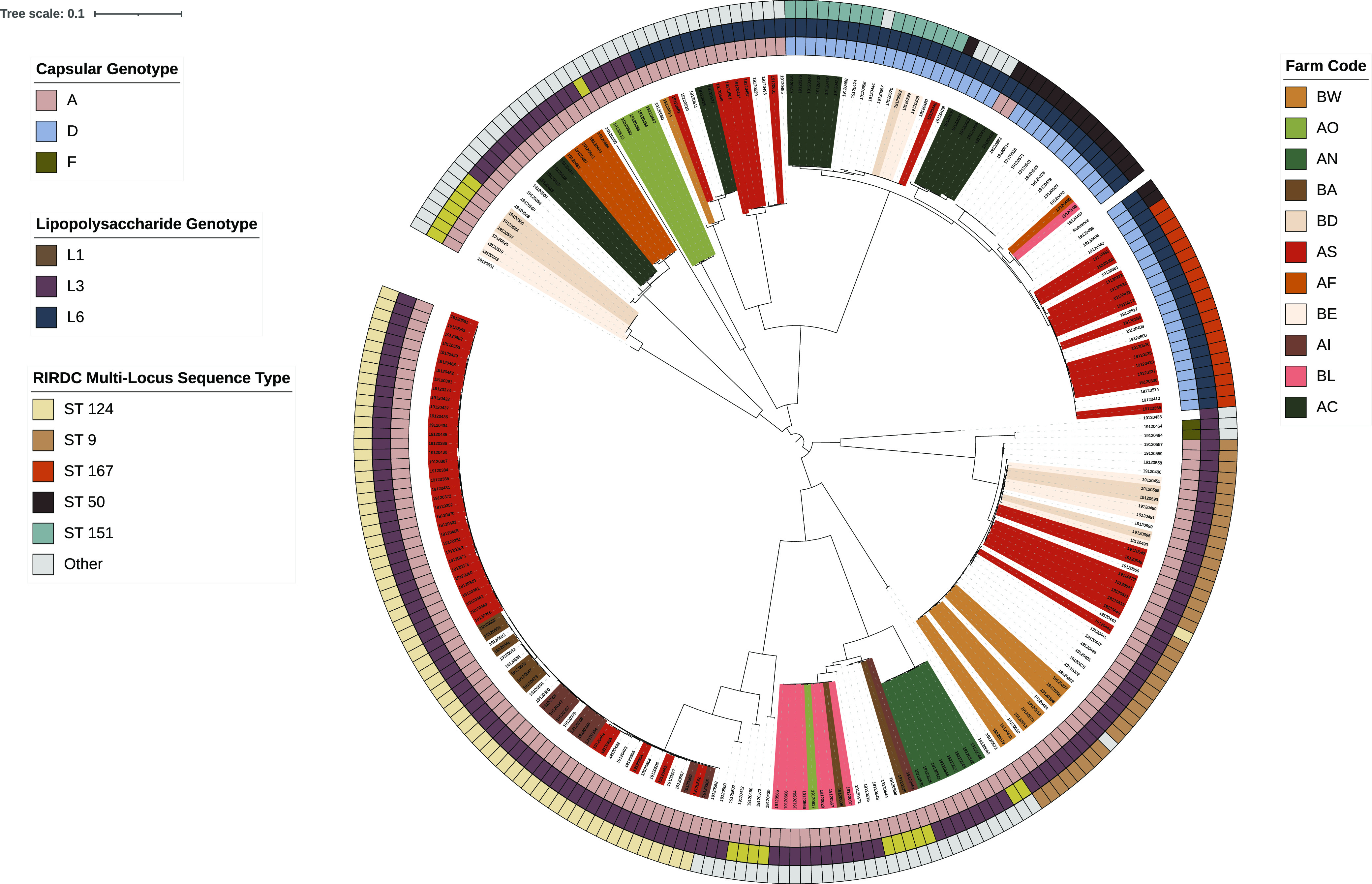
Phylogenetic tree of 242 Pasteurella multocida isolates from Australian pigs with respiratory disease, highlighting the major RIRDC STs along with their respective capsular and LPS genotypes and farm of origin. Farms contributing >5 isolates had their branch labels colored by farm code. The order of annotations from the inner ring to the outer ring is as follows: capsular genotype, LPS genotype, and RIRDC MLST ST.

Core genome phylogenetic analysis of the 242 viable isolates revealed clustering based on STs except for a single ST 124 isolate, which grouped with all ST 9 isolates (Fig. 1). Clustering was seen among STs 167, 50, and 151, while STs 124 and 9 appeared to be genetically distinct. Within each of the major STs (181 of the 252 total isolates), isolates were typically of the same capsular and LPS genotypes; only 2 isolates differed from the dominant capsular genotype of other isolates in their ST, and only 5 isolates differed from the dominant LPS genotype of other isolates in their ST.

**FIG 2:**
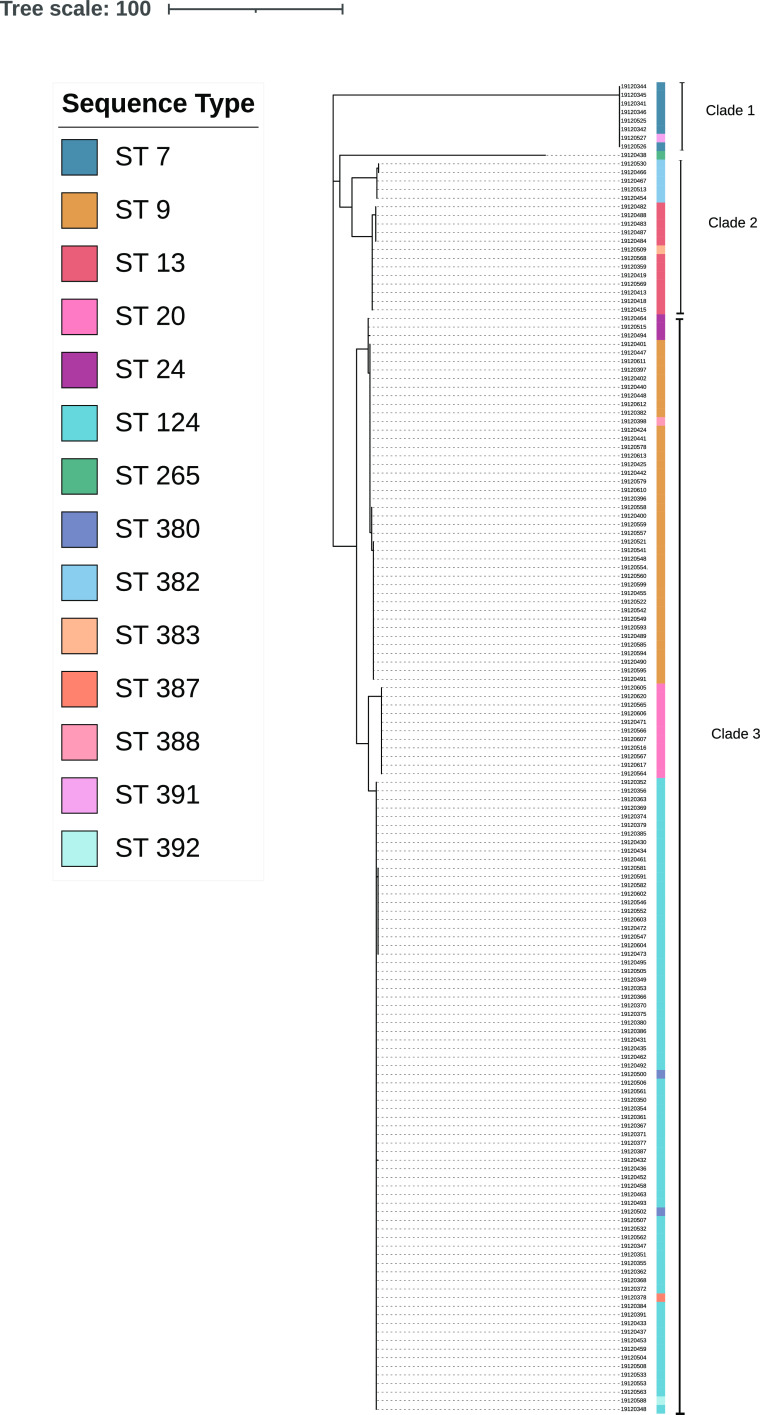
Phylogenetic tree of 155 L3 genotype Pasteurella multocida isolates from Australian pigs with respiratory disease, highlighting intrastrain variations within this LPS genotype.

Subsequent phylogenetic analysis of each of the represented LPS genotypes (genotypes L1, L3, and L6) identified various degrees of intra-LPS-genotype diversity. Among the 155 L3 genotype isolates, three main clades were evident. Clade 2 and clade 3, while clearly diverging, displayed more moderate similarity to each other than to clade 1, for which the differences were far more obvious (Fig. 2). The 20 L1 genotype isolates were broadly separated into three main clades, with clear distinctions between clade 1 and its clade 2 and clade 3 counterparts, for which the difference was less evident (see Fig. S1). The 76 L6 genotype isolates demonstrated a much lower level of intra-LPS-genotype diversity (see Fig. S2).

### Strain-based trends.

All of the major STs were identified on multiple farms across Australia (Fig. 1). Isolates belonging to ST 124 were identified on 9 farms, ST 9 was detected on 13 farms, ST 167 was detected on 7 farms, ST 50 was detected on 14 farms, ST 20 was detected on 8 farms, and ST 151 was detected on 11 farms. These strains were also isolated over several years; ST 124, ST 9, and ST 50 isolates were identified every year of the studied time period (2014 to 2019), ST 20 isolates were identified in all studied years except 2014, ST 167 isolates were identified in all studied years except 2016 and 2019, and ST 151 isolates were identified in all studied years except 2017 and 2019. In regard to the site of isolation, all non-respiratory tract-derived isolates except 1 were found to belong to the same dominant STs (ST 124, ST 9, ST 50, and ST 151) as respiratory tract-derived isolates.

All of the detected strains were also examined for association with identified AMR genotypes (Table 4). Isolates in the collection that were carrying ARGs belonged to nine AMR genotypes, harboring between 1 and 8 genes. Except for ST 167, for which 19 of its 20 isolates shared a single AMR genotype, no other affiliations between STs and AMR genotypes were apparent. Due to the high frequency of virulence factor carriage across all strains, affiliations of specific virulence factors with specific strains were difficult to identify.

**TABLE 4:** AMR genotype detection rates and associated RIRDC STs for 252 Pasteurella multocida isolates collected from Australian pigs between 2014 and 2019

AMR genotype^*a*^	Detection rate (%)	ST (no. of isolates)
8 genes: *ant(9)-Ia*, *aph(3′)-Ia*, *aph(6)-Id*, *bla*_ROB-1_, *dfrA14*, *erm*(A), *sul2*, *tet*(B)	1.2	9 (3)
5 genes: *aph(3″)-Ib*, *aph(3′)-Ia*, *aph(6)-Id*, *sul2*, *tet(Y)*	7.5	167 (19)
4 genes: *aph(3′)-Ia*, *aph(6)-Id*, *dfrA14*, *sul2*	0.4	50 (1)
4 genes: *aph(3″)-Ib*, *aph(3′)-Ia*, *aph(6)-Id*, *sul2*	0.4	151 (1)
3 genes: *aph(3″)-Ib*, *sul2*, *tet(B)*	2.4	151 (6)
2 genes: *ant(9)-Ia*, *erm(A)*	6.3	9 (15), 388 (1)
1 gene: *bla*_ROB-1_	0.4	20 (1)
1 gene: *floR*	0.4	7 (1)
1 gene: *tet(B)*	5.6	9 (2), 50 (2), 124 (1), 185 (1), 384 (1), 389 (3), 13 (4)
0 genes	75.5	7 (6), 9 (19), 11 (2), 12 (5), 13 (8), 18 (2), 20 (16), 24 (3), 37 (1), 50 (16), 58 (7), 124 (69), 141 (1), 151 (10), 167 (1), 185 (2), 265 (1), 379 (5), 380 (2), 381 (1), 382 (5), 383 (1), 385 (1), 387 (1), 389 (1), 390 (2), 391 (1), 392 (1)

a The number of ARGs identified is indicated for each genotype.

## DISCUSSION

Despite the assortment of virulence factors detected with high frequency among the isolates in this study, our analysis of Australian porcine P. multocida strains highlighted several vulnerabilities that can be exploited to reduce the impact on industry.

The low frequency of detection of AMR means that a number of antimicrobials (especially those of low importance in human medicine) remain viable options for treating P. multocida infections in Australian pigs when necessary. Respiratory infections are commonly managed using an array of antimicrobials, including β-lactams, macrolides, phenicols, potentiated sulfonamides, and tetracyclines (17). Resistance rates of <1% were detected for antimicrobials representing the first four of those five drug classes, accompanied by an arguably low rate of resistance to tetracyclines (23%). Neomycin has no clinical breakpoints for P. multocida and thus could be assessed only based on European Committee on Antimicrobial Susceptibility Testing (EUCAST) epidemiological cutoff (ECOFF) values, which indicated that 9.2% of isolates were non-wild-type strains. No resistance to any antimicrobials designated by the World Health Organisation as highest-priority critically important antimicrobials (ceftiofur, ciprofloxacin, gamithromycin, tilmicosin, or tulathromycin) (18) or as high importance by the Australian Strategic and Technical Advisory Group on AMR (ASTAG) (ceftiofur or ciprofloxacin) (16) was detected, and no multidrug resistance was detected.

Although not entirely congruent with the antimicrobial susceptibility data, the high degree of phenotypic susceptibility to antimicrobials agreed with the low incidence of ARG carriage (75.5% with no AMR genes). Two of the nine detected AMR genotypes involved genes conveying resistance to three or more antimicrobial classes; however, the failure to translate to phenotypic resistance, perhaps due to gene downregulation or lack of expression, meant that the strains were not classified as multiclass resistant. Additionally, the AMR genotypes identified in this study showed limited affiliation with a particular ST, with the majority of dominant strains seen exhibiting several different AMR genotypes, indicating that factors other than AMR account for their population dominance. An exception was ST 167, for which 19 of the 20 isolates detected over a 4-year period shared the same AMR genotype, harboring five ARGs. The degree of genetic stability displayed by ST 167 indicates an ability to retain ARGs despite various selective pressures and flags it as particularly deserving of continued monitoring. The antimicrobial susceptibility data from this study indicate that several antimicrobials that are commonly relied upon for the treatment of P. multocida infections in Australian piggeries should remain efficacious.

In comparing the results from various studies, allowance should be made for the possibility of variations in sampling design and laboratory methods affecting the observed differences. Nevertheless, there are some similarities and differences concerning this study and others that are notable. Comparison of the P. multocida AMRs screened for in both this study and a previous Australian study (12) showed either a decrease in frequency of detection (ampicillin from 4% to 0.4%, florfenicol from 2% to 0.4%, trimethoprim-sulfamethoxazole from 2% to 0.8, and tetracycline from 28% to 22.5%) or maintenance of 0% detection across both studies (ceftiofur, tilmicosin, and tulathromycin). Overall resistance rates were also low in comparison with recent international studies from Brazil (19), China (20), the Czech Republic (21), Europe (22), South Korea (23), Spain (24), Taiwan (25), North America (26), and Vietnam (27). Australia had the lowest (or equal lowest) rates of resistance for all tested antimicrobials in this study with available comparisons except for tetracycline, with a higher rate of resistance (23.3%) than those in Spain (18.8%) and Europe (20.4%), and florfenicol, with a higher rate of resistance (0.4%) than those in China (0%) and the United States and Canada (0%) (see Table S4 in the supplemental material). The higher rate of tetracycline resistance could be explained by its ASTAG ranking as a low-importance antimicrobial, along with its broad-spectrum activity, making it an attractive choice for first-line treatment in Australian pigs (16).

Expanding further on international comparisons, all available P. multocida isolates in PubMLST (https://pubmlst.org) pertaining to porcine respiratory infections were screened for commonality with the major STs identified in the present study. Based on this comparison, ST 167 is unique to Australian pigs. ST 20, while unique to Australia, has historically been isolated from fowl cholera cases, with a high affinity for poultry. ST 124 has only one recorded incidence of detection outside Australia, from a sheep in New Zealand (1995). ST 50 has been detected in pigs in Spain (2002 and 2003), China (2003, 2020, and 2021), the Czech Republic (2001 to 2004), Denmark (2006), India (2007 to 2009 and 2015), and South Korea (2016). ST 50 was also isolated from a turkey in the United Kingdom (1997) and a rabbit in Italy (2003, 2004, 2010, and 2015). ST 151 has been detected in Vietnam (1999). ST 9 has been detected in poultry from Australia (1988, 1992, and 2016 to 2019) and Denmark (2006), rabbits from Italy (2010, 2011, and 2015) and the Czech Republic (2001), pigs in Spain (2003 and 2004) and China (2021), and a cow in India (1992). The NCBI genome database for P. multocida (https://www.ncbi.nlm.nih.gov/genome/browse#!/prokaryotes/pasteurella) contains 60 whole-genome-sequenced pig-derived isolates, 47 of which were derived from a single study on respiratory isolates; only 4 of those were of an ST (ST 9) detected in the present study (9). The identification of common STs between Australia and other countries and host species suggests the occurrence of exchange events enabling the dissemination of these strains, although it may be difficult to pinpoint in which direction those events occurred. Although the Australian porcine P. multocida population has demonstrated a degree of overlap with international strains, the high level of representation of STs detected only in Australia highlights a distinctly local genetic identity, likely a by-product of Australia’s unique isolated geography and restrictions on the importation of livestock (28). The other points to be highlighted here include the apparent lack of host discrimination by infecting P. multocida strains, particularly between poultry and pigs, as also noted in a previous Australian study (10), and the biosecurity implications that accompany this finding.

Vaccination to prevent respiratory disease in pigs involving P. multocida is attractive for convenience of management, animal welfare, and avoidance of antimicrobial use. Historic vaccination efforts have often centered on the LPS component due to its role as a major immunogen, often serving as the target of protective antibodies produced by the host’s immune response (29), although those efforts have seen various degrees of success (30). A 2013 study seeking to further elucidate differences among P. multocida LPS structures found that the heterogeneity extended beyond the interstrain variations, with intrastrain variations being found among 23 isolates belonging to the L3 LPS genotype (29). The present study examined a larger collection of 155 L3 isolates, along with 20 L1 and 76 L6 genotype isolates, and observed intrastrain variation within all three genotypes. It is possible that the intrastrain variation within LPS genotypes may be a contributing factor to the low efficacy of previous LPS-centric vaccines. Comparing isolates based on their entire genome, with particular emphasis on isolate MLST ST, LPS genotype, and capsular genotype, can inform the design of more targeted vaccines with potentially greater efficacy.

### Conclusion.

Our limited knowledge of P. multocida strains from lower respiratory tract disease in pigs in Australia has now been increased by a modern surveillance process based on high-throughput robotics. The data showed that genetically distinct Australian P. multocida strains were highly represented, and only low levels of AMR were found, compared to isolates from other countries. This approach to surveillance based on large-scale exploitation of standard assays promises to deliver timely advice, yielding practical gains in disease management and antimicrobial stewardship. Animal health programs could more broadly benefit from a wider adoption of this approach and extension beyond the scope of porcine P. multocida.

## MATERIALS AND METHODS

### Isolates tested.

The study utilized a collection of 252 porcine P. multocida isolates that were obtained over a 5-year period (2014 to 2019) by ACE Laboratory Services, a major provider of veterinary diagnostic services to the Australian pig industry. The isolates originated from 66 farms across Australia and were predominantly derived from the lower respiratory tract of diseased pigs at postmortem examinations (Table 1). Because non-respiratory tract-derived isolates were present in the collection, they were included in the study for comparison.

Isolates that were confirmed to be P. multocida by matrix-assisted laser desorption ionization–time of flight (MALDI-TOF) mass spectrometry (Bruker) were subcultured overnight at 37°C on sheep blood agar (MM1120; Edwards) and then stored in brain heart infusion broth with 20% glycerol (CM1135; Oxoid) at −80°C prior to downstream testing.

### Antimicrobial susceptibility testing.

Antimicrobial susceptibility testing of all 252 P. multocida isolates was performed using the RASP methodology (31). The approach involved estimation of MICs by broth microdilution assay involving 12 antimicrobials, i.e., ampicillin, ceftiofur, chlortetracycline, ciprofloxacin, florfenicol, gamithromycin, gentamicin, neomycin, tetracycline, tilmicosin, trimethoprim-sulfamethoxazole, and tulathromycin. Clinical and Laboratory Standards Institute (CLSI) clinical breakpoints (32) were used for interpretation of resistance where available, and EUCAST ECOFF values (https://mic.eucast.org) were used in their absence. The RASP methodology allows this number of MIC assays to be all completed in a 5-day period (from culture to susceptibility testing, including drug plate preparation), with data electronically captured for pairing with genomic results and reporting.

### Genomic sequencing and analysis.

All P. multocida isolates were subjected to WGS using an Illumina NextSeq sequencer and Celero library preparation kits (Tecan). DNA extraction was performed using the MagMAX multisample DNA extraction kit (Thermo Fisher Scientific) according to the manufacturer’s instructions. DNA library preparation was performed using the Celero library preparation kit according to the manufacturer’s protocol. DNA libraries were sequenced on the Illumina NextSeq platform using a 300-cycle midoutput v2 reagent kit. Isolate sequences were assembled using SPAdes v3.14.0 (33) before undergoing the following analyses: ABRicate v1.0.1 (34) was used with a custom database containing genes allowing screening for virulence factors and capsular and LPS genotyping (see Table S1 in the supplemental material for accession numbers), ARGs were identified using ResFinder (35), MLST determination was performed using mlst v2.19.0 (36), and isolates with previously unidentified STs (37 across the RIRDC MLST and multihost MLST P. multocida typing schemes) were registered in the PubMLST database (https://pubmlst.org/organisms/pasteurella-multocida). All isolates were assigned both a multihost ST and a RIRDC ST but, in the interest of simplicity, only the RIRDC MLST scheme is referred to in this paper.

Phylogenetic trees were generated based on 242 isolate fastq files using single-nucleotide polymorphisms (SNPs) identified within the core genome with Snippy v4.1.0 (37), followed by recombination removal with ClonalFrameML v1.11 (38). Maximum likelihood phylogenetic trees were constructed in RAxML v0.9.0 (39) using the generalized time-reversible model set at 1,000 bootstraps and were visualized in iTOL (40). The primary phylogenetic tree compared all 242 isolates, while subsequent trees compared only isolates of the same LPS genotype.
