# Supplementary material for: Antimicrobial Resistance of and Genomic Insights into Pasteurella multocida Strains Isolated from Australian Pigs
Source: Microbiol Spectr. 2023 Jan 18;11(1):e03784-22. doi: 10.1128/spectrum.03784-22 (PMC9927299; doi:10.1128/spectrum.03784-22)
Supplement: Supplemental file 1 — Tables S1 to S4 and Fig. S1 and S2. Download spectrum.03784-22-s0001.pdf, PDF file, 0.3 MB [file spectrum.03784-22-s0001.pdf]

Supplementary Tables

Table S1 | *Pasteurella multocida* genes and accession numbers used in custom Abricate database.

Gene	Gene function	Description	Reference
kmt-1	Integral component of membrane	Identification of all *P. multocida* isolates	CP017961.1
exbB	Iron acquisition	Accessory protein Ton-dependent transport of iron compounds	CP006976
exbD	Iron acquisition	Accessory protein Ton-dependent transport of iron compounds	AE004439.1
fimA	Adhesins	Fimbriae	AE004439.1
fur	Iron acquisition	Ferric uptake regulation protein	AF027154.1
hgbA	Iron acquisition	Haemoglobin-binding protein	CP006976
hgbB	Iron acquisition	Haemoglobin-binding protein	CP001409.1
hsf-1	Adhesins	Autotransporter adhesion	AE004439.1
hsf-2	Adhesins	Autotransporter adhesion	CP006976.1
nanB	Sialidases	Outer membrane-associated proteins – an autotransporter protein	CP097791.1
nanH	Sialidases	Outer membrane-associated proteins – small sialidases	CP097622.1
oma87	Protectins	Outer membrane protein 87	AE004439.1
ompA	Protectins	Outer membrane protein A	CP006976
ompH	Protectins	Outer membrane protein H	CP004392.1
pfhA	Adhesins	Filamentous haemagglutinin	AY035342.1
plpB	Protectins	Lipoprotein B	CP007205.2
pmHAS	Hyaluronidase	Hyaluronan synthase	CP006976.1
ptfA	Adhesins	Filamentous hemagglutinin	CP006976.1
soda	Superoxide dismutase	Superoxide dismutase	LR134488.1
sodC	Superoxide dismutase	Superoxide dismutase	AE004439.1
tadD	Adhesins	Putative nonspecific tight adherence protein D	CP006976.1
tbpA	Iron acquisition		CP006976.1
tonB	Iron acquisition	Iron transporters, transport ferric-siderophore complexes	CP097608.1
toxA	Toxin	Dermonecrotic toxin	AJ566110.1
hyaD-hyaC	Capsular serotype A		AF067175
bcbD	Capsular serotype B		AF169324
dcbF	Capsular serotype D		AF302465
ecbJ	Capsular serotype E		AF302466
fcbD	Capsular serotype F		AF302467
pcgD	Lipopolysaccharide genotype L1		MT542700.1
nctA	Lipopolysaccharide genotype L2		GQ444331.1
gatF	Lipopolysaccharide genotype L3		KF314826.1
latB	Lipopolysaccharide genotype L4		KM670447.1
rmlA	Lipopolysaccharide genotype L5		JN571483.1
nctB	Lipopolysaccharide genotype L6		KJ689443.1

ppgB	Lipopolysaccharide genotype L7		JX987238.1
natG	Lipopolysaccharide genotype L8		KM670448.1

Table S2 | Carriage of antimicrobial resistance genes by 252 Australian pig *Pasteurella multocida.*

Gene	Class	Subclass	% Detected
ant(9)-Ia_1	Aminoglycoside	Spectinomycin	7.5
aph(3'')-Ib_2	Aminoglycoside	Streptomycin	10.3
aph(3')-Ia_3	Aminoglycoside	Kanamycin	9.5
aph(6)-Id_1	Aminoglycoside	Streptomycin	9.5
blaROB-1_1	β-lactam	Cephalosporin	1.6
dfrA14_5	Folate Pathway Antagonist	Trimethoprim	1.6
erm(A)_1	Macrolide	-	7.5
floR_2	Phenicol	Chloramphenicol	0.4
sul2_2	Folate Pathway Antagonist	Sulphonamide	9.9
sul2_9	Folate Pathway Antagonist	Sulphonamide	2.0
tet(B)_1	Tetracycline	Tetracycline	6.3
tet(B)_2	Tetracycline	Tetracycline	2.8
tet(Y)_1	Tetracycline	Tetracycline	7.5

Table S3 | RIRDC_MLST *Pasteurella multocida* strain prevalence of 252 Australian pig isolates.

RIRDC_ST	Count	Prevalence (%)
124	70	27.8
9	39	15.5
167	20	7.9
50	19	7.5
20	17	6.7
151	17	6.7
13	12	4.8
7	7	2.8
58	7	2.8
12	5	2.0
379	5	2.0
382	5	2.0
389	4	1.6
24	3	1.2
185	3	1.2
11	2	0.8

18	2	0.8
380	2	0.8
390	2	0.8
37	1	0.4
141	1	0.4
265	1	0.4
381	1	0.4
383	1	0.4
384	1	0.4
385	1	0.4
387	1	0.4
388	1	0.4
391	1	0.4
392	1	0.4

Table S4 | International comparison of antimicrobial resistant *Pasteurella multocida* from pigs with respiratory infections.

Country	Year	n_Isolates	% R_Amo	% R_Amp	% R_Cef	% R_Cip	% R_Ctx	% R_Ctc	% R_Dox	% R_Ery	% R_Enr	% R_Ffn	% R_Gam
Australia (QLD)	1995	60		0	-		-	-		3		-	-
Australia (NSW, QLD, WA, SA and VIC)	2002 - 2013	51		4	0		-	-		14		2	-
Australia	2014 - 2019	266		0.4	0	0	-	22.9		-		0.4	0
Brazil	2016	40	0		22.5		-			40	22.5		
China	2003 - 2007	233	80.3		0	0		65.2		6		0	
Czech Republic	2007 - 2011	332	45.5	6	0.6		-				1.5	1.5	
Europe	2009 - 2012	152			0		-				0	0.7	
Korea	2010 - 2016	454		4.8	0.2		-	36.8			2.6	18.5	
Spain	2017 - 2018	32		40.6			-			12.5	0		
Taiwan	2013 - 2015	62					-		87.1	100		91.9	
US and Canada	2011 - 2015	855		2	0		-				0	0	
Vietnam	2011 - 2018	83	75.9	9.6			0			9.6	0		

Supplementary Figures

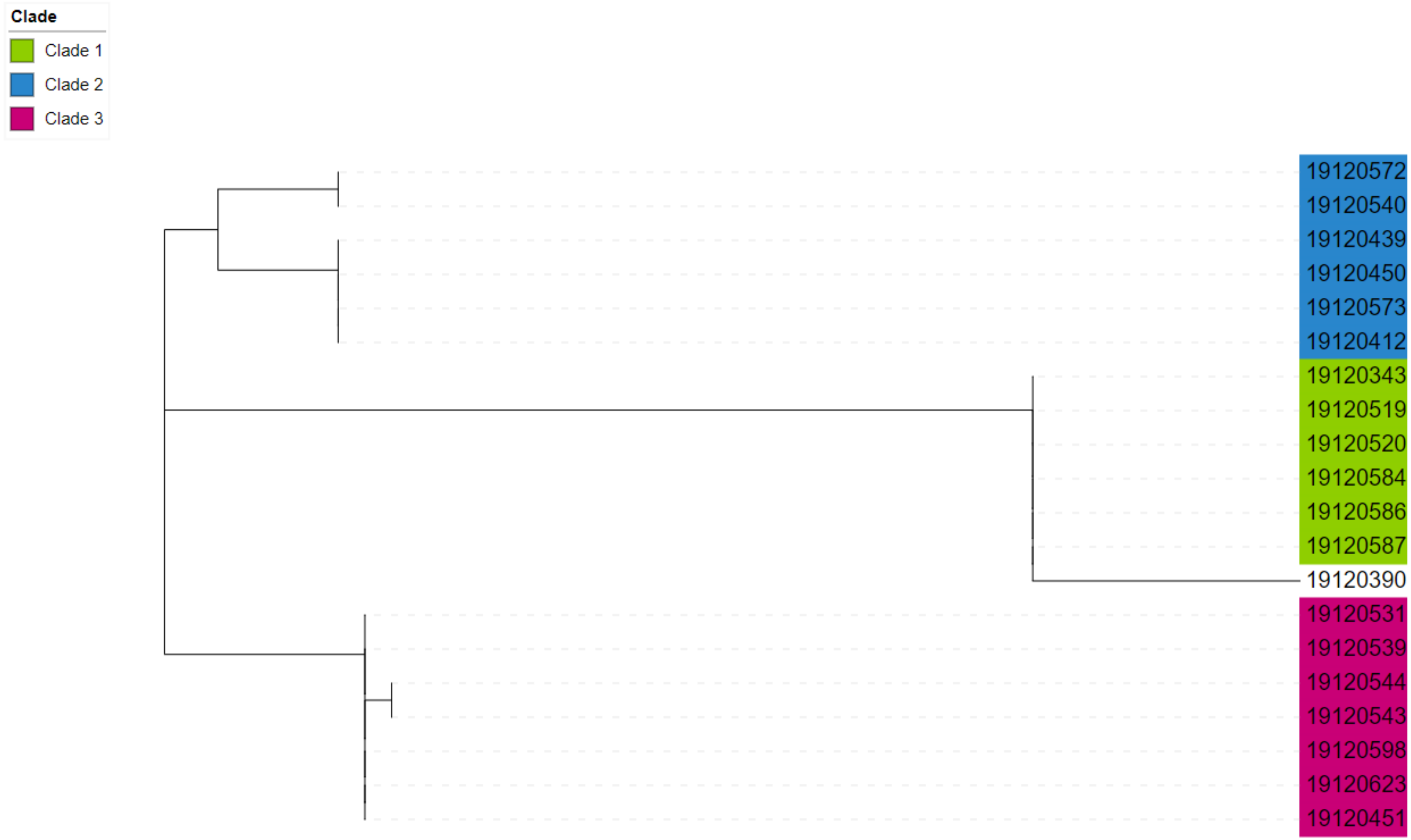

Figure S1 | Phylogenetic tree of 20 L1 genotype Pasteurella multocida isolates from Australian pigs with respiratory disease highlighting intra-strain variation within this lipopolysaccharide genotype.

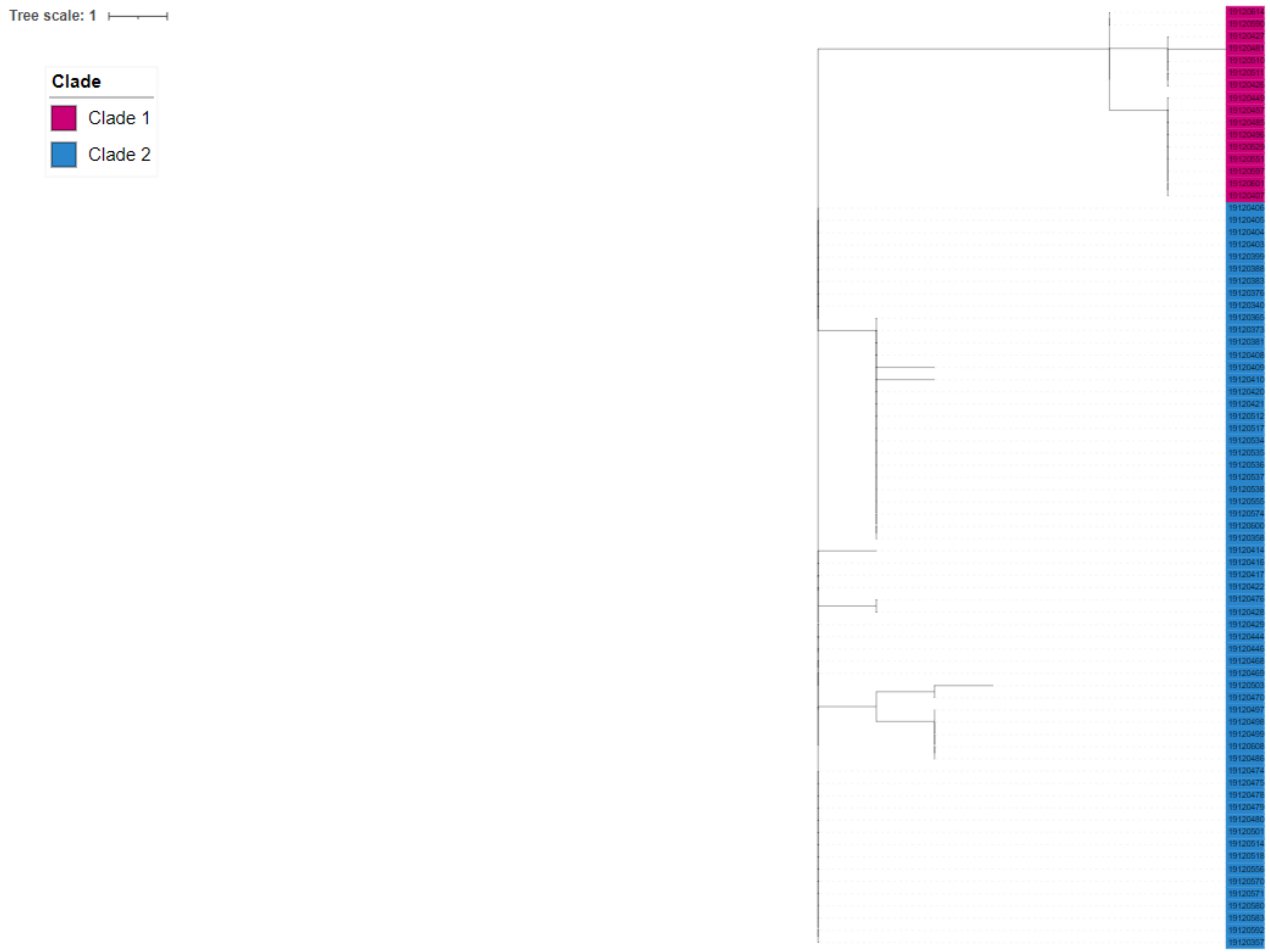

Figure S2 | Phylogenetic tree of 76 L6 genotype Pasteurella multocida isolates from Australian pigs with respiratory disease highlighting intra-strain variation within this lipopolysaccharide genotype.
